# Global trends in oncology research: A mixed‐methods study of publications and clinical trials from 2010 to 2019

**DOI:** 10.1002/cnr2.1650

**Published:** 2022-06-11

**Authors:** Vincent Akiki, Xavier Troussard, Jean‐Philippe Metges, Patrick Devos

**Affiliations:** ^1^ Comité National de Coordination de la Recherche Paris France; ^2^ Department of Haematology CHU Caen Normandie Caen France; ^3^ CHU de Brest Inserm, University Brest, EFS Brest France; ^4^ Univ Lille, CHU Lille, Lillometrics France

**Keywords:** bibliometrics, cancer, clinical trials, publications, research assessment

## Abstract

**Purpose:**

To evaluate the cancer research effort of some major countries over two 5‐year periods (2010–2014 and 2015–2019) on the basis of scientific publications and interventional clinical trial metrics and to analyze the relationship between research effort and cancer burden (incidence and mortality).

**Materials and Methods:**

Clinical trials were extracted from ClinicalTrials.gov using a specific query. Publications were identified in Web of Science (WoS) using a query based on keywords and were then analyzed using InCites, a bibliometric tool. Bibliometric indicators were computed per country and per period.

**Results:**

During 2010–2019, 1 120 821 cancer‐related publications were identified in WoS, with 447 900 and 672 921 (+50%) articles respectively published in 2010–2014 and 2015–2019. Meanwhile, 38% and 7% of the articles were published in oncology and cell biology journals, respectively. Exactly 30% of the published articles were contributed by the USA. In the study period, China strongly increased its production and overspecialization. Apart from China, which had a low normalized citation impact (NCI), almost all countries increased their NCIs; in particular, France's NCI increased from 1.69 to 2.44. As for clinical trials, over 36 856 were opened worldwide during that period. Over 17 000 (46.5%) opened in the USA, which remained the leader during the study period. China ranked second worldwide in terms of the number of open trials in 2015–2019. Results revealed that the 17 cancer localizations versus cancer burden and research effort showed no evident relationship.

**Conclusion:**

The results may provide a scientific basis for decision making for continued research. Based on bibliometric data, this type of study will aid public health policymaking and lead to a more transparent public fund allocation.

## 1 BACKGROUND

Cancer is a major public health problem throughout the world. In fact, reports from the World Health Organization showed approximately 9.6 million cancer‐related deaths in 2018, or about one in six deaths, thus making cancer the second leading cause of mortality worldwide.^1^ In the same year, the number of new cases was estimated at 18.1 million.^2^, ^3^ Cancer is also a pathology that affects many organs. In this regard, there are many types of mortality depending on the affected organs, gender, socioeconomic factors, and geographic area. The economic impacts of cancer are also substantial, with global costs estimated at 1.2 billion dollars in 2010.^4^

Individual nations have established unique structures dedicated to the care of cancer patients. These structures have also resulted in numerous research programs targeted at cancer diagnosis and management. While many data are available on the prevalence, incidence, and mortality rate of cancer,^3^, ^5^, ^6^ there is a lack of information concerning the research activities carried out in oncology. In 2019, approximately 60 000 articles were published in oncology journals across the world, thus putting oncology in eighth place among all disciplines and first among the medical disciplines (Web of Science (WoS)/Incites data). In this regard, many robust databases are available for finding and retrieving scientific publications, with notable resources including PubMed, WoS (Clarivate Analytics), Scopus (Elsevier), and CrossRef. These databases are further reinforced by analytical tools such as InCites (Clarivate Analytics) and SciVal (Elsevier), which enable researchers to conduct bibliometric analyses on the basis of factors such as the specific discipline (e.g., oncology, neurology), country of origin, and affiliated institution. However, from 1995 to 2019, only about 250 bibliometric studies related to cancer are shown in PubMed. These include studies describing the 50 or 100 most cited articles in a given cancer site or articles describing the evolution of the number of publications about a given cancer type. These studies are generally limited to a single cancer^7^, ^8^, ^9^ or to a single country of origin.^10^ In fact, none of the available studies measured scientific output at the global level, by country of origin, or for all cancers combined. Addressing this gap is the objective of the current work.

In order to guarantee the transparency of results, the International Committee of Medical Journal Editors requires that all clinical studies be declared in an international registry prior to initiation.^11^ Here, researchers may use the ClinicalTrials.gov database, which was established in 2000,^12^ specifically to declare the main parts of all relevant studies. As such, the database provides access to information on clinical trials initiated around the world, thus making it possible to measure research efforts in a given pathology based on the country of origin. In this study, the main objective of gathering information on scientific publications and interventional clinical trials was to measure the cancer research efforts (quantitative and qualitative data) of different countries over two separate five‐year periods, namely, 2010–2014 and 2015–2019. A five‐year period is used in many bibliometric indicators and academic and research‐related institution ranking, such as Shanghai Ranking^13^ or SCImago.^14^

We searched the two following databases:

- The WoS database and InCites platform, which provide volumes of publications per country and individual article impacts in terms of the number of received citations;
- The ClinicalTrials database, which provides information related to clinical trial involvement.

Analyses were first performed for all cancers combined and then by individual location while distinguishing between hematologic and solid tumors. This essentially constituted an analysis of all cancers combined.

## 2 METHODS

Data on publications were extracted from the WoS,^15^ which is marketed by Clarivate Analytics. The WoS categorizes journals based on their stated disciplines (e.g., neuroscience, surgery, or immunology)^16^; the oncology category therefore consists of specific oncology journals. Previous internal analyses conducted on these WoS categories have revealed that only one‐third of all cancer publications have been published in oncology journals, as oncology is a very transversal discipline, while the remaining two‐thirds have been published in specialty journals (e.g., neurology, pneumology, or urology). In order to count all publications, we used the following criteria for our search query:

- Published in a cancer journal;
- Contains cancer‐generic keywords in the title (Title field in the WoS) or author keywords (Author Keywords field in the WoS); examples include “cancer,” “tumor,” and “neoplasms”;
- Contains specific localization cancer keywords in the title or author keywords; examples include “glioma,” “glioblastoma,” and “mesothelioma.”

We identified appropriate keywords for our searches by using terms available in the “Medical Subject Headings,” a controlled vocabulary thesaurus used in Medline for indexing articles,^17^ and by selecting those found in a sample of articles published in oncology journals. The resulting list was subsequently validated by two oncology experts, Pr. X. Troussard from CHU Caen Normandie^18^, ^19^ and Pr. JP Metges from CHU de Brest.^20^, ^21^ We then examined the contributions from major countries with reference to the global output, as determined based on the number of articles with authors from each country. We also compared the production of different countries with reference to their overall production in biomedical research (Medical & Health Science in the WoS database) and citation impact. The following two bibliometric indicators were also computed:

- The specialization index (SI), which is the ratio of the percentage of articles from a given country related to specific areas of disease to the percentage of articles concerning the same areas of disease worldwide. Index values >1 indicate that the given country has an overspecialization in that disease area while index values <1 indicate underspecialization. The SI is a robust indicator with which we can observe the general changes in the research profile of a country over time.^22^
- The category normalized citation impact (CNCI or NCI); for a given article, the CNCI is the ratio of the observed number of citations to the expected number of citations (defined as the mean number of citations for all articles published in the same year in the same WoS category). Here, a normalized bibliometric indicator compensates for the fact that the number of citations a given article receives is partly influenced by the year of publication (i.e., older articles have more opportunities for citation) and size of the scientific field.^23^ The CNCI of a given country is the mean CNCI of all articles co‐authored by that country; the benchmark value is 1, with higher CNCI values reflecting higher impacts in terms of citation numbers.

Next, we conducted our bibliometric analysis using the InCites analytical tool,^24^ which was developed by Clarivate Analytics based on data indexed in the WoS. In this study, we only analyzed documents that were categorized as “Article” or “Review” in the WoS. We then extracted clinical trial data from ClinicalTrials.gov, specifically considering all interventional studies in cancer (Condition = Cancer) that were initiated from 2010 to 2019 across the world. The field “Country” identified the different countries participating in each study while the “Conditions” field made it possible to reclassify studies according to the different types of investigated cancer. Finally, the “Funder” field was used to determine whether studies were promoted by an academic institution or industry.

## 3 RESULTS

### 3.1 Scientific publications

For the period of 2010–2019, we found a total of 1 120 821 cancer‐related publications in the WoS, including 447 900 from 2010–2014 and 672 921 from 2015–2019, reflecting an increase of just over 50%. Table 1 shows the number of publications by discipline (WoS categories). From 2015 to 2019, only 38.2% of publications were found in oncology journals. Excluding cell biology (+2.1%), experimental medicine (+2.2%), and hematology (−1.3%), the distributions changed slightly between the two periods in terms of discipline.

**TABLE 1:** First 25 Web of Science categories of cancer articles from 2010 to 2019

		2010–2014		2015–2019	
Web of Science Category	Total	Number of Publications		Number of Publications	
All articles	1 120 821	447 900	Part (%)	672 921	Part (%)
Oncology	425 926	169 130	37.8	256 796	38.2
Cell Biology	80 100	26 328	5.9	53 772	8.0
Surgery	76 285	34 591	7.7	41 694	6.2
Medicine, Research, & Experimental	67 585	21 024	4.7	46 561	6.9
Biochemistry & Molecular Biology	67 146	28 591	6.4	38 555	5.7
Radiology, Nuclear Medicine, & Medical Imaging	61 237	26 428	5.9	34 809	5.2
Pharmacology & Pharmacy	57 152	22 554	5.0	34 598	5.1
Hematology	51 872	24 347	5.4	27 525	4.1
Pathology	47 811	20 917	4.7	26 894	4.0
General & Internal Medicine	43 815	13 276	3.0	30 539	4.5
Gastroenterology & Hepatology	39 489	17 673	3.9	21 816	3.2
Multidisciplinary Sciences	38 782	15 612	3.5	23 170	3.4
Immunology	36 822	16 212	3.6	20 610	3.1
Genetics & Heredity	27 840	12 580	2.8	15 260	2.3
Clinical Neurology	27 310	12 124	2.7	15 186	2.3
Urology & Nephrology	27 258	11 857	2.6	15 401	2.3
Obstetrics & Gynecology	25 017	11 224	2.5	13 793	2.0
Biotechnology & Applied Microbiology	23 214	9167	2.0	14 047	2.1
Public, Environmental, & Occupational Health	22 711	10 143	2.3	12 568	1.9
Chemistry, Multidisciplinary	21 080	6168	1.4	14 912	2.2
Endocrinology & Metabolism	18 376	8239	1.8	10 137	1.5
Chemistry, Medicinal	18 354	7819	1.7	10 535	1.6
Respiratory System	16 943	7139	1.6	9804	1.5
Pediatrics	15 267	7194	1.6	8073	1.2
Biophysics	14 536	6417	1.4	8119	1.2

*Note*: List of the first 25 Web of Science categories in which cancer articles have been published.

Table 2 shows the number of publications and ranks for both investigated periods, specifically concerning the 20 countries with the highest scientific production rates in the area of cancer from 2010 to 2019 (WoS documents retrieved through the query). These data were compared with all medical research (Medical & Health Sciences) and documents published in oncology journals. As such, we found that the largest contribution was provided by the United States for both periods while China's contribution increased from fourth to second for all medical disciplines and remained stable in the second position for cancer during both periods. Japan and South Korea were also better positioned in oncology than in medical research at large. Conversely, the United Kingdom was better positioned for medical research at large than for cancer research. This suggests the existence of overspecialization in China, Japan, and South Korea and underspecialization in the United Kingdom. The percentage of articles published by each country in oncology journals is mostly close to the world average of 43.2% (484 298/1 120 821), except a few countries with lower rates, including Brazil with 28.9% (5806/20096), India with 34.0% (11 695/34402), and Turkey with 34.9% (7435/21278); and a few countries with higher rates, including Sweden with 48.5% (9449/19493), Canada with 48.5% (21 806/44954), the Netherlands with 48.9% (15 815/32365), and Belgium with 50.4% (7681/15229).

**TABLE 2:** World ranking for cancer‐related biomedical research and cancer‐related articles in oncology journals

	Cancer	Medical & health sciences				Cancer documents in the Web of Science				Oncology journals			
	2010–2019	2010–2014		2015–2019		2010–2014		2015–2019		2010–2014		2015–2019	
Country	Nb docs	Nb docs	Rank	Nb docs	Rank	Nb docs	Rank	Nb docs	Rank	Nb docs	Rank	Nb docs	Rank
World	1 120 821	2 599 256		3 206 267		447 900		672 921		200 810		283 488	
USA	334 212	856 893	1	997 732	1	145 541	1	188 671	1	71 278	1	86 687	1
China	233 415	182 821	4	398 470	2	66 373	2	167 042	2	29 595	2	81 117	2
Japan	82 898	147 206	5	169 870	5	36 144	3	46 754	3	15 969	3	20 007	3
Germany	75 024	183 806	3	208 346	4	33 595	4	41 429	4	14 400	4	17 743	4
Italy	68 160	133 040	6	162 590	6	28 583	6	39 577	5	12 569	6	16 764	5
UK	67 289	222 553	2	269 254	3	29 073	5	38 216	6	13 356	5	16 100	6
France	51 652	112 053	8	128 922	9	22 905	7	28 747	7	10 596	7	13 177	7
South Korea	48 028	81 807	13	105 690	13	19 717	8	28 311	8	7622	9	11 003	9
Canada	44 954	130 399	7	161 455	7	19 061	9	25 893	9	9696	8	12 110	8
India	34 402	86 077	11	117 868	10	10 442	13	23 960	10	4545	13	7150	13
Spain	33 567	85 991	12	108 092	11	14 012	10	19 555	12	5727	12	8045	12
Australia	32 910	105 142	9	147 331	8	13 085	12	19 825	11	6209	11	8571	11
Netherlands	32 365	87 212	10	106 754	12	13 889	11	18 476	13	6947	10	8868	10
Taiwan	22 711	39 323	19	44 639	20	9993	14	12 718	15	3709	16	5535	14
Turkey	21 278	64 793	15	79 798	15	7980	16	13 298	14	3675	17	3760	18
Brazil	20 096	81 597	14	100 636	14	7730	18	12 366	16	2230	20	3576	20
Sweden	19 493	51 175	17	65 025	17	8437	15	11 056	18	4314	14	5135	16
Switzerland	19 179	54 771	16	71 512	16	7848	17	11 331	17	3765	15	5217	15
Poland	16 582	34 704	20	46 585	19	6530	19	10 052	19	2621	19	3690	19
Belgium	15 229	40 300	18	50 776	18	6339	20	8890	20	3252	18	4429	17

Abbreviations: Nb docs, number of documents (articles and reviews) published; Rank, world ranking for cancer‐related biomedical research (medical & health sciences) and cancer‐related articles in oncology journals (20 main countries).

Notice that the number of publications in oncology journals is not exactly the same between Tables 1 and 2. This is due to the fact that publications from multidisciplinary journals may be reclassified into specific subject areas in Incites.

**FIGURE 1:**
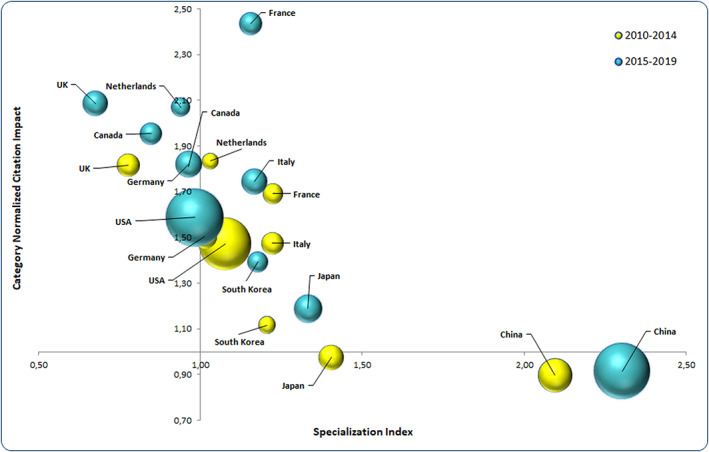
CNCI and SI evolution of the top 10 countries with the highest numbers of cancer‐related publications. Evolution between 2010–2014 and 2015–2019 regarding the normalized citation impact (CNCI) and specialization index (SI) for the 10 countries with the highest numbers of cancer‐related publications. Bubble sizes are correlated with the number of publications

Figure 1 shows the 10 countries with the highest outputs in oncology journals over the entire investigated period as well as the evolution of their specialization indices (national over‐ and underspecializations shown for given fields) and normalized impact indices (citation impacts) between the subdivided periods. First, China clearly showed a strong overspecialization (which increased again between the two periods), along with somewhat lesser overspecializations in Japan, Italy, South Korea, and France. There was also a notable underspecialization in the United Kingdom. Apart from China, which currently shows a low CNCI, almost all countries increased their CNCIs. This was particularly evident in France, which showed an increase from 1.69 to 2.44.

### 3.2 Clinical trials

**FIGURE 2:**
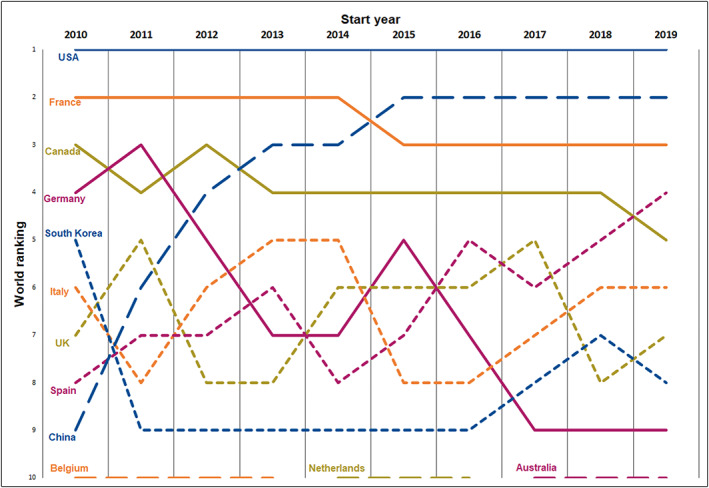
Top 10 country rankings in terms of the number of cancer‐related interventional clinical trials. Evolution of top 10 countries' yearly rankings in terms of the number of cancer‐related interventional clinical trials from 2010 to 2019

A total of 175 309 interventional studies were initiated worldwide from 2010 to 2019, and these include 36 856 cancer‐related studies; a comparison of the years 2010 and 2019 showed 2848 and 4571 articles, respectively. This overall percentage of around 20% remained stable over the 10‐year investigated period. Of the 36 856 total studies, 1% received US federal funding, 11% received funding from the National Institute of Health, 38% received industrial funding, and 75% received “other funding” (a single study could also receive co‐funding). The “other” category mainly referred to studies that were promoted by academic institutions (e.g., hospitals, universities) or research groups (e.g., the European Organization for Research and Treatment of Cancer, Brussels, Belgium). Table 3 shows the 20 countries with the highest number of interventional cancer studies initiated over the investigated period while Figure 2 shows the global rankings. With more than 17 000 studies, the United States participated in 46.5% of all such studies initiated worldwide and remained the leader over the entire period. It was followed by China, which showed a strong increase, specifically rising from ninth place in the world in 2010 to second place in 2019, thus surpassing France, which nevertheless moved up to third place. We also observed a strong progression in Spain, which exceeded Canada in 2019. Conversely, Germany dropped from fourth to ninth. Further, the national rankings differed significantly when categorized based on funding; for example, Spain sharply increased its rank in industrial studies while China and France sharply increased their ranks in academic studies.^25^

**TABLE 3:** Top 20 most prominent countries in terms of cancer‐related clinical trials

Country	TOTAL	2010	2011	2012	2013	2014	2015	2016	2017	2018	2019	Part (%)	% Evol 2010/2019^a^
GLOBAL	36 856	2848	2961	3033	3129	3468	3887	4131	4327	4501	4571		+60
USA	17 148	1536	1487	1495	1535	1643	1776	1753	2003	2016	1904	46.5	+24
China	4759	154	214	260	302	348	503	661	676	764	877	12.9	+469
France	3730	284	322	344	364	374	415	404	401	422	400	10.1	+41
Canada	2857	259	248	275	274	304	291	328	326	302	250	7.8	−3
Spain	2501	154	208	224	249	260	272	280	314	285	255	6.8	+66
Germany	2444	214	272	246	241	261	288	252	253	242	175	6.6	−18
UK	2403	176	214	216	241	266	272	255	324	244	195	6.5	+11
Italy	2392	184	202	232	256	266	265	235	283	262	207	6.5	+13
South Korea	2169	187	186	192	211	233	258	211	263	253	175	5.9	−6
Belgium	1614	129	141	154	192	170	170	147	187	185	139	4.4	+8
Netherlands	1505	116	124	126	156	183	188	158	164	158	132	4.1	+14
Australia	1467	98	108	115	138	149	165	133	207	192	162	4.0	+65
Japan	1153	92	94	97	107	114	138	117	151	128	115	3.1	+25
Taiwan	1073	69	70	81	91	135	127	110	140	142	108	2.9	+57
Poland	1047	82	98	93	107	110	118	102	128	109	100	2.8	+22
Denmark	927	59	79	62	102	104	113	98	116	108	86	2.5	+46
Israel	877	65	71	69	103	92	95	94	105	98	85	2.4	+31
Russia	844	71	102	75	84	70	91	74	103	90	84	2.3	+18
Brazil	735	57	77	65	59	70	80	60	101	86	80	2.0	+40
Austria	688	66	66	71	77	73	79	64	71	63	58	1.9	−12

*Note*: List of the 20 most prominent countries classified on the basis of the number of cancer‐related clinical trials initiated from 2010 to 2019.

^a^ “% Evol 2010/2019” = [(Number of publications in 2019/Number of publications in 2010) − 1] × 100.

### 3.3 Relationship between the burden of cancer localization and research production

Table 4 shows the number of new cases and deaths in 2018 for the main cancers at the global level as well as the numbers of clinical trials and publications from 2015 to 2019. There are some immediately noticeable differences in the rankings for epidemiological data (incidence and mortality) and research efforts (clinical trials and publications). For one, breast cancer showed the highest incidence rate, with intermediate mortality (sixth rank) and high research efforts, while hematology ranked first in terms of research but substantially lower in terms of incidence (sixth) and mortality (fifth). In addition, esophageal and gastric cancers ranked fourth and second in terms of incidence and mortality but ranked eighth and tenth in terms of research, respectively.

**TABLE 4:** Incidence versus mortality versus opened trials versus publications

	Incidence		Mortality		Trials		Publications	
	2018		2018		2015–2019		2015–2019	
Cancer localization	Nb cases	Rank	Nb cases	Rank	Nb	Rank	Nb	Rank
Breast	2 261 419	1	684 996	6	2595	2	64 046	2
Lung	2 237 641	2	1 822 422	1	2227	3	50 522	3
Colorectal	1 931 590	3	935 173	3	1510	4	38 229	4
Esophagus & Stomach	1 693 203	4	1 312 869	2	976	8	27 339	10
Prostate	1 414 259	5	375 304	10	1209	5	27 909	8
Hematology	1 278 362	6	711 840	5	3490	1	103 772	1
Uterus	1 021 494	7	439 201	9	618	13	8672	16
Head & Neck	931 931	8	467 125	7	947	9	27 534	9
Liver	905 677	9	830 180	4	768	11	28 566	7
Thyroid	586 202	10	43 646	16	208	16	10 531	14
Bladder	573 278	11	212 536	12	419	14	9931	15
Pancreas	495 773	12	466 003	8	772	10	15 694	12
Kidney	431 288	13	179 368	14	403	15	11 127	13
Skin	324 635	14	57 043	15	1116	6	37 884	5
Ovary	313 959	15	207 252	13	679	12	15 779	11
Brain	308 102	16	251 329	11	1009	7	37 596	6
Testis	74 458	17	9334	17	32	17	2147	17

*Note*: Incidence (number of cases in 2018), mortality (number of cases in 2018), number of opened trials (2015 to 2019), number of publications (2015 to 2019) and ranks of 17 cancer localizations.

## 4 DISCUSSION

By finding both the number of publications and number of registered clinical trials, this study explored the breadth of cancer research at the global level and based on the different types of cancer. At the global level, we found discrepancies between the burdens of cancer by incidence and mortality rates as well as in research production. Although Miao,^7^ Akmal,^8^ and Andersen^9^ used similar methodologies, their investigations were restricted to scientific production rates for esophagogastric cancer, glioblastoma, and hematology. As our searches were based on specific keyword queries, the number of returned publications might not have been exhaustive. However, our objective was to compare different countries over two unique periods, in which case any potential bias should not imply a significant difference in rankings. In fact, the proportion of publications found in oncology journals (nearly 38%) confirmed that our decision to conduct a keyword analysis was appropriate. By querying the ClinicalTrials.gov database, we were also able to observe a rapid increase for China and Spain regarding their participation in cancer‐related clinical trials. Unfortunately, inclusion data are not available through ClinicalTrials.gov; the figures also presented centers without inclusion. Further, only interventional studies were included as many other types of studies (e.g., registries or population‐based studies) may be conducted without registration.

The United States played a leading role in cancer research in the 10‐year investigated period, accounting for around 30% of all related publications and 46% of clinical trials. However, major developments are evident in other countries over the same period. For example, China made significant progress, as shown by its SI (>2). In Japan, an intense and persistent overspecialization might have been catalyzed due to the fallout from Hiroshima and Nagasaki, with sustaining factors, including a voluntary policy targeted at esophageal cancer screening, which has been implemented for more than 20 years, and a generally high incidence of cancer (more than one million new cases in 2018). Meanwhile, the United Kingdom showed underspecialization (decreasing over the second period of investigation) and increasing NCI, both of which are surprising in view of its cancer incidence rate (similar to those found in Japan, France, and Italy) and high level of scientific production in biomedical research. The results are similar when looking at clinical trials. Finally, it should be noted that France is well‐positioned in terms of the citation impact of scientific publications (NCI rose from 1.69 to 2.44) and clinical trials (third in the world). These advancements may partly be explained based on the implementation of three successive cancer plans (from 2003 to 2019) and substantial provisions of dedicated resources.

We also found a strong correlation between the number of registered trials and number of publications. There was only a weak correlation between epidemiological data (incidence and mortality) and research efforts (trials and publications). This may partly be explained by the different prevalence rates for certain cancers between countries (e.g., high rates of breast cancer in the United States, colorectal cancer in Japan, and prostate cancer in the United Kingdom). Further, cancer research is not coordinated at the global level, meaning that individual countries tend to focus on different cancers. Carter^26^ and Coronado^27^ evaluated the relationship between research funding and societal burdens at several cancer sites in the United Kingdom and Canada and showed various rates of over‐ and underfunding after adjusting for mortality and life expectancy.

## 5 CONCLUSIONS

The data herein could offer general value and constitute a scientific basis for deciding which areas of focus are most appropriate in continued research at the continental, national, and regional levels, specifically in locations with high rates of mortality. Based on bibliometric data, this type of study should also contribute to the formation of public health policies while encouraging the more transparent allocation of public and associated types of funding.

## AUTHOR CONTRIBUTIONS

**Vincent Akiki:** Data curation (equal); formal analysis (equal); methodology (equal); visualization (equal); writing – review and editing (equal). **Xavier Troussard:** Validation (equal); writing – review and editing (equal). **Jean‐Philippe Metges:** Validation (equal); writing – review and editing (equal). **Patrick Devos:** Conceptualization (equal); formal analysis (equal); methodology (equal); project administration (equal); supervision (lead); visualization (equal); writing – original draft (equal); writing – review and editing (equal).

## FUNDING INFORMATION

This research did not receive any specific grant from funding agencies in the public, commercial, or not‐for‐profit sectors.

## CONFLICT OF INTEREST

The authors have stated explicitly that there are no conflicts of interest in connection with this article.

## ETHICS STATEMENT

This study is a review of data from ClinicalTrials.gov and Web of Science. It does not require ethics approval.

## Data Availability

The data that support the findings of this study are available from the corresponding author upon reasonable request.
